# Multimodal retrieval of autobiographical memories: sensory information contributes differently to the recollection of events

**DOI:** 10.3389/fpsyg.2015.01681

**Published:** 2015-11-05

**Authors:** Johan Willander, Sverker Sikström, Kristina Karlsson

**Affiliations:** ^1^Department of Social Work and Psychology, University of Gävle Gävle, Sweden; ^2^Gösta Ekman Laboratory, Department of Psychology, Stockholm UniversityStockholm, Sweden; ^3^Department of Psychology, Lund UniversityLund, Sweden; ^4^Department of Psychology, Stockholm UniversityStockholm, Sweden

**Keywords:** autobiographical memory, multimodal, multisensory, unimodal, age distribution, experiential ratings

## Abstract

Previous studies on autobiographical memory have focused on unimodal retrieval cues (i.e., cues pertaining to one modality). However, from an ecological perspective multimodal cues (i.e., cues pertaining to several modalities) are highly important to investigate. In the present study we investigated age distributions and experiential ratings of autobiographical memories retrieved with unimodal and multimodal cues. Sixty-two participants were randomized to one of four cue-conditions: visual, olfactory, auditory, or multimodal. The results showed that the peak of the distributions depends on the modality of the retrieval cue. The results indicated that multimodal retrieval seemed to be driven by visual and auditory information to a larger extent and to a lesser extent by olfactory information. Finally, no differences were observed in the number of retrieved memories or experiential ratings across the four cue-conditions.

## Introduction

Autobiographical memory has been defined as personal events from one’s life (Conway and Pleydell-Pearce, 2000). These personally experienced memories may be evoked by various cues (e.g., photographs). Previous research investigating cued retrieval of autobiographical memories has mostly focused on unimodal cues and studies on multimodal cueing of autobiographical memory are scarce (Karlsson et al., 2013).

In the unimodal paradigm, participants are presented with a cue of a single modality and asked to retrieve an autobiographical memory related to the cue. However, in everyday life individuals are exposed to combinations of sensory inputs originating from several modalities simultaneously (e.g., vision, audition, and olfaction). Therefore the overall aim of the present study was to investigate multimodal cueing of autobiographical memories, i.e., cues pertaining to several modalities simultaneously. More specifically, we investigated these memories with respect to the age distribution and experiential ratings (e.g., valence, the feeling of being brought back in time to the occurrence of the event). In addition modelling was carried out to determine the relative contribution of visual, olfactory, and auditory sensory information in multimodal retrieval of autobiographical memories.

A well-established finding in the autobiographical memory literature is the age distribution (Rubin and Schulkind, 1997). It has consistently been shown that the frequency distribution of autobiographical memories across the lifespan follows a specific pattern that comprises three components, i.e., the childhood amnesia, the bump, and recency (Rubin and Schulkind, 1997; Conway and Pleydell-Pearce, 2000; Bruce et al., 2005). The childhood amnesia reflects the inability to retrieve memories that occurred prior to three to 4 years of age (Rubin et al., 1998; Conway and Pleydell-Pearce, 2000). The childhood amnesia is succeeded by the bump period reflecting a peak in the age distribution typically located to 10–30 years for verbally and visually evoked memories (Rubin et al., 1998; Conway and Pleydell-Pearce, 2000; Willander and Larsson, 2006; for a review see Koppel and Berntsen, 2015). Likewise, the age distribution for memories evoked by musical cues peak between 10–20 years (Schulkind and Woldorf, 2005). However, for olfactory evoked memories a different pattern emerges, where the bump is located before 10 years of age (Chu and Downes, 2000; Willander and Larsson, 2006, 2007, 2008; Larsson et al., 2014; cf. Rubin et al., 1984).

Memories evoked by verbal, visual, or olfactory cues differ also with respect to experiential ratings. For example, it has been demonstrated that odor-evoked memories are rated as more pleasant than memories evoked by words (Chu and Downes, 2002; Herz and Schooler, 2002; Willander and Larsson, 2007). Herz (2004) also demonstrated that odor-evoked memories are experienced as more pleasant than memories evoked by pictures or sounds. In contrast, when individuals are asked to retroactively rate the emotionality experienced at the occurrence of the event, picture-evoked events are reported as more emotional than word- and odor-evoked memories (Willander and Larsson, 2006; cf. Ehrlichman and Bastone, 1992).

Differences in the experiential ratings of events as a function of cue-modality have also been observed on mental time travel (i.e., the feeling of being brought back in time to the occurrence of the event). Herz and Schooler (2002) demonstrated that memories evoked by odors are associated with a stronger feeling of being brought back than memories evoked by verbal cues. This finding has been replicated and extended in two experiments by Willander and Larsson (2006, 2007), where odor-evoked memories were found to be associated with a stronger feeling of being brought back than memories evoked by words or pictures.

Another important experiential dimension in autobio raphical memory recollection is vividness; although the results are somewhat mixed. For example, Goddard et al. (2005) found that verbal cues result in more vividly recollected events compared to pictorial or olfactory evoked events. However, neither Herz (2004) nor Willander and Larsson (2006, 2007) were able to detect any differences in vividness of retrieved autobiographical events across cue-modality (i.e., words, pictures, or odors).

In the autobiographical memory literature, sensory infor ation from different modalities are treated as separate entities rather than integrated multimodal representations. However, a growing body of research on multisensory perception indicates that unimodal information may be integrated into multimodal representations (e.g., Driver and Spence, 2000). Although unimodal perceptual information may be integrated, the relative influence of the respective modalities may differ. A well-established finding in perception is that the visual modality dominates over other modalities (e.g., Colavita and Weisberg, 1979; Koppen and Spence, 2007). For example, Colavita and Weisberg (1979) demonstrated that individuals were more prone to respond to the termination of visual (light) stimuli compared to auditory stimuli, even though these modalities were presented simultaneously.

Few studies have yet addressed multimodal cueing of events in autobiographical memory. Willander and Larsson (2007) indirectly addressed bimodal cues when presenting participants with odors in conjunction with their respective names. Here it was found that when odors were presented with its corresponding name, the age distribution and experiential ratings took an intermediate position compared to the unimodal cueing conditions (Willander and Larsson, 2007). However, it may be argued that a word/semantic cue does not adequately reflect or fully represent the sensory information perceived at the occurrence of an event since individuals rarely perceive sensory information in conjunction with its corresponding verbal label. In a study by Karlsson et al. (2013), the semantic representation of autobiographical memories evoked by multimodal cues were contrasted with events triggered by unimodal cues. In this study the analyses were based on the verbal reports of the retrieved autobiographical memories of the same data set as the current study. The aim of Karlsson et al. (2013) was to highlight multimodal retrieval of events from a content perspective rather than a distributional perspective like the current study. The results of Karlsson et al. (2013) study indicated that the semantic representation of multimodally evoked memories could be described as a combination of the three unimodal conditions (visual, olfactory, and auditory). Furthermore, the visual and auditory conditions contributed more than the olfactory condition to the semantic representation of the multimodal condition. This finding provided further support for the notion of a modality hierarchy (Karlsson et al., 2013).

The aim of the present study was to investigate naturalistic multimodal cues, and compare them with corresponding unimodal cues presented in the visual, auditory, and odor modalities, in the context of autobiographical memory retrieval. The following hypotheses were made: based on previous studies on the age distribution of autobiographical memories, the bump of odor-evoked events should be located earlier (i.e., to the first decade of life) than the bump of visually and auditory evoked events. Also, the multimodal age distribution should fall in between the unimodal distributions, and the relative weight of the odor distribution should be smaller than the auditory and visual distributions. With regard to experiential ratings, it is hypothesized that odor-evoked memories are rated as more pleasant and associated with a stronger feeling of being brought back as compared the to visual and auditory conditions. The unimodal conditions should not differ with regard to vividness and importance.

## Materials and Methods

### Participants

Sixty-two students (see **Table 1** for participant information) at the Department of Psychology, Stockholm University, took part in the study for course credits. All participants provided informed consents. The current project was approved by the Ethical Committee Stockholm (EPN Stockholm).

**Table 1:** Participant information across the four conditions.

	Total	Visual	Auditory	Olfactory	Multimodal
*n*_total_	62	17	16	13	16
*n*_women_	46	13	12	10	11
*n*_men_	16	4	4	3	5
Mean age years (SD)	23.94 (2.70)	23.53 (2.67)	23.56 (1.90)	24.62 (2.84)	24.19 (3.35)
Age range years	20–30	20–29	20–29	20–30	20–30


### Design

The design was a four-way between subjects design, where the participants were randomly assigned to one of four conditions (three unimodal or one multimodal).

### Materials

The stimuli materials consisted of 15 pictures, 15 odors, and 15 sounds (see Appendix A for the respective cues). In the unimodal conditions all cues were presented separately whereas in the multimodal condition cues from the three unimodal conditions were presented simultaneously. The unimodal cues were selected so that they could be combined into a multimodal naturalistic context (see Appendix A). Pilot testing resulted in 15 contexts and their unimodal constituents. For example, the context *harbor* was represented by a photo of a harbor by the sea containing fishing boats, sounds from fishing boats, sea birds, sea waves, and the smell of fish. The photographs were real world scenes depicting the visual environment of the context. The sounds consisted of real world sounds typical of the contexts. Finally, the olfactory stimuli consisted of odors typical for the contexts. The olfactory cues consisted of non-synthethic odors, which were kept in non-translucent glass jars to prevent visual inspection.

The multimodal cues were constructed by combining pictures, odors, and sounds from the three unimodal conditions in such a way that all unimodal cues came from the same natural context.

### Experiential Ratings

Retrieved memories were rated on the following experiential dimensions: (1) How strong is your feeling of being brought back to the occurrence of the event? (2) How emotional do you experience the event? (3) How pleasant do you experience the event? (4) How important do you experience the event (5) How vivid is your memory of the event? For question 1, 2, 4, and 5, a 5-point a Likert scale was used, where 1 = not at all, 5 = very much. For question 3, a 5-point a Likert scale was used, where 1 = very unpleasant, 5 = very pleasant.

### Procedure

All subjects were tested individually and were given the following instruction: *You will be presented with a number of memory cues. Your task is to try to remember specific events related to the respective cues. The event may have taken place at any time in your life. Once you remember an event please describe the event verbally as detailed as possible (if possible provide sensory information, feelings and so on). You will be given three minutes to describe the event verbally. You will also be asked to rate the event on some phenomenological dimensions.*

Thirty seconds per cue were allowed for retrieval. Given that the participant were able to generate a memory, three minutes were devoted for the verbal description, which was recorded with a digital audio recorder. The presentation order of the 15 cues was randomized for each participant. For the unimodal conditions the cues were administered in the following way: In the visual condition the pictures were presented on a 22-inch LCD computer screen. Auditory cues were presented with a pair of AKG 701 reference headphones connected to the same computer controlling the visual presentation. The odors were provided in non-translucent glass jars. The participants held the jars themselves and were given a cue to signal the start of sniffing. In the multimodal condition, the cues were presented simultaneously and in the same way as in the respective unimodal conditions.

Each event was rated on the five experiential dimensions, as specified in the *experiential ratings* section, immediately after the verbal description. Lastly, following the retrieval phase all memories were dated according to the age of the participants at the occurrence of the events.

## Results

### Number of Evoked Memories

The number of evoked memories for each participant were analyzed using a one-way ANOVA with modality, i.e., visual (*M* = 11.53, *SD* = 3.50), auditory (*M* = 9.81, *SD* = 2.93), olfactory (*M* = 9.00, *SD* = 3.21), and multimodal (*M* = 10.88, *SD* = 3.20), as between-subject factor. No main effect of modality was observed [*F*(3,58) = 1.81, *p* > 0.05, η^2^ = 0.085].

### Age Distributions

In order to statistically analyze the age distributions, the lifespan was segmented into six 5-year intervals, (i.e., 0–5, 6–10, 11–15, 16–20, 21–25, and 26–30 years). Next, the proportions of memories in each interval for the respective participants were calculated by dividing the number of memories in a particular interval with the total number of retrieved events for that participant. The proportions of memories were then analyzed using four separate one-way ANOVAs (i.e., visual, auditory, olfactory, and multimodal) with interval as within group factor. Given that the age distributions were analyzed with four separate ANOVAs the *p*-level was Bonferroni adjusted (i.e., *p* = 0.05/4).

First, for the visual age distribution Mauchly’s test of sphericity indicated unequal variances across intervals and thus the ANOVA was Greenhouse–Geisser corrected. The ANOVA indicated a significant main effect of interval [*F*(2.62,41.97) = 6.24, *p* < 0.01, η^2^ = 0.28]. Repeated contrasts (SPSS 21) indicated that the proportion of memories in the 6–10 year interval was higher than in the 0–5 year interval, the 16–20 year interval contained a higher proportion of memories than 11–15 (*p* < 0.05), and the 21–25 year interval contained a higher proportion of memories than the 26–30 year interval. No other repeated contrasts were significant (*p*s > 0.05). The participants of the four conditions retrieved between 4–15 memories. However, in the visual condition one of the participants only retrieved two memories, therefore an additional ANOVA without this participant was computed. The results did not change following the exclusion of the participant with only two memories [*F*(2.59,38.81) = 5.19, *p* < 0.01, η^2^ = 0.26].

For the auditory age distribution Mauchly’s test of sphericity indicated unequal variances across intervals, consequently the ANOVA was Greenhouse–Geisser corrected. The ANOVA indicated a significant main effect of interval [*F*(3.00,44.96) = 5.58*, p* < 0.01, η^2^ = 0.27]. Repeated contrasts indicated that the proportion of memories in the 6–10 year interval was higher than in the 0–5 year interval and the proportion of memories was higher in the 21–25 year interval compared to the 26–30 year interval (*p*s < 0.05). No other comparisons were significant (*p*s > 0.05).

For the olfactory condition the ANOVA indicated a marginally significant main effect of interval [*F*(5,60) = 2.76, *p* = 0.026, η^2^ = 0.19]. Repeated contrasts indicated that the 6–10 year interval contained a higher proportion of memories than the 0–5 year interval and the 11–15 year interval (*p*s < 0.05). No other comparisons were significant (*p*s > 0.05).

Finally, for the multimodal age distribution Mauchly’s test of sphericity indicated unequal variances across intervals. Therefore, the ANOVA was Greenhouse–Geisser corrected. No significant effect of interval was observed [*F*(2.80,75) = 2.53*, p* = 0.074, η^2^ = 0.14].

**FIGURE 1:**
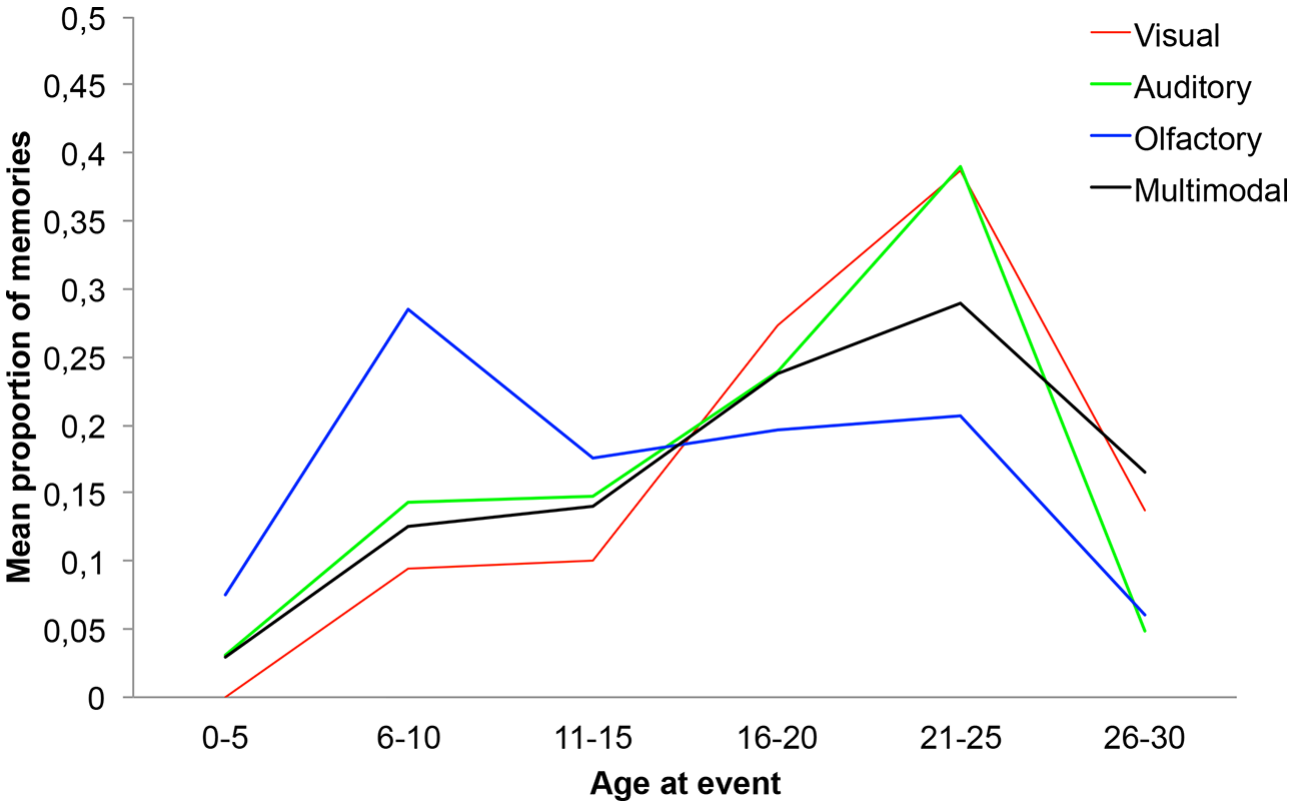
**The age distributions of the four cue-conditions**.

The age distributions are displayed in **Figure 1**.

### Decomposing the Age at Event Distribution into the Early Life Bumps and Forgetting

Conceptually the autobiographical memory distribution can also be divided into an early life bump, which in previous literature typically has been found to occur around 10–30 years at the event (Koppel and Berntsen, 2015), and a recency gradient that presumably occurs due to more recent memories are less forgotten compared to older ones. Typically, these two components can be observed in age at event data; however, more recently Janssen et al. (2011) suggested a method where these two components can be decomposed through a mathematical procedure. In the present study we investigated how cues of different sensory modalities quantitatively impact on the bump and the forgetting curve components of autobiographical memories. We studied this by applying the Janssen et al. (2011) procedure to our data.

This procedure consists of six steps that are carefully described in Janssen et al. (2011), and here we briefly summarize the steps; (1) The proportion of events are calculate for each participant separately and (2) a power-function is fitted to all participants based on the 10 most recent years. (3) A predicted value of the forgetting curve is calculated for each participant, using the fitted value from step (2) and empirical data from step (1). (4) The result from step (1) is divided with the results from step (3). (5) The resulting distribution from step (4) is normalized for each participant and (6) averaged over all participants. This procedure was conducted separately for each of the four conditions in our dataset.

#### The Autobiographical Memory Bump

**FIGURE 2:**
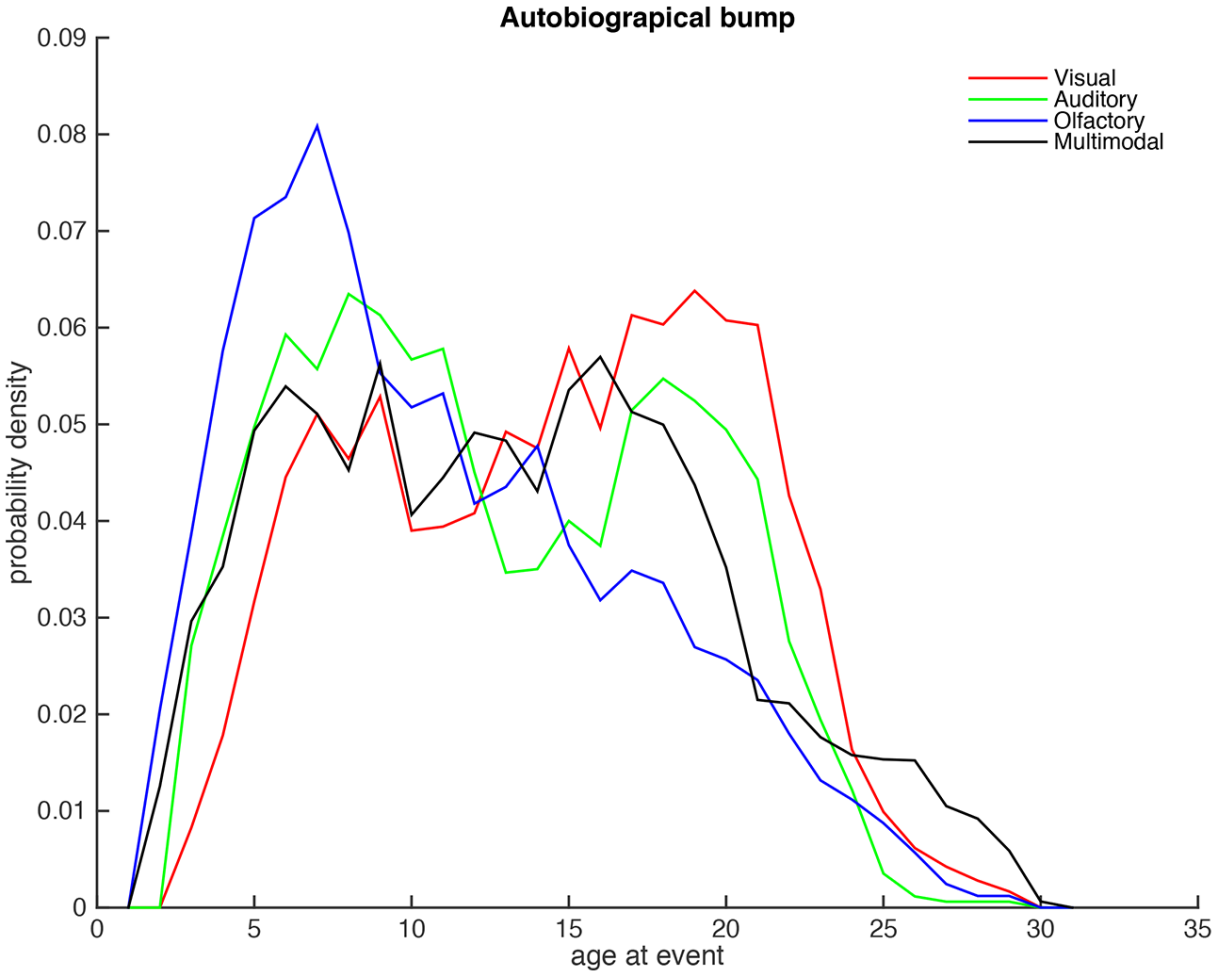
**The corrected autobiographical memory distributions for the four conditions**.

**Figure 2** shows the resulting probability density distributions, where the originally data is smoothed with a moving average methods consisting of 5 years. In essence this figure shows the age at event distributions when the forgetting component is mathematically removed from the dataset. Several interesting findings can be noted from this analysis. First, the perhaps most apparent results is that olfactory distribution (blue) has a peak around six years of age that is earlier than the visual and auditory modalities. Second, in contrast, the auditory and visual modalities has two peaks, an early peak around eight, which appears to be slightly later than the olfactory peak, and a later peak around the age of 20 that is absent in the olfactory modality. Third, although, the distributions of visual and auditory age at events are similar, the early auditory peak is larger than later auditory peaks, whereas the later visual peak is larger than the early visual peak. Fourth, the multimodal peak is more similar to the visual and auditory modalities, than the olfactory modality; however, the multimodal condition does not have the pronounced peaks in the interval from five to twenty years of the events.

To further measure the similarity of the age at event density distributions, we calculated the dot products between the vector representing this distribution (i.e., the length of each distribution was first normalized to one, and two distributions where multiplied and summed, and finally the square root of the resulting value was presented). The results (see **Table 2**) show that visual and the auditory distributions are more similar, whereas the olfactory distribution is more dissimilar to these distributions. In contrast, the multimodal distribution is approximately as similar to the three unimodal conditions.

**Table 2:** The dot product between the densities of the age at event distributions for the four modalities.

	Visual	Auditory	Olfactory	Multimodal
Visual	–	0.9769	0.9237	0.9719
Auditory		–	0.9728	0.9823
Olfactory			–	0.9711
Multimodal				–


#### Forgetting Curves

**FIGURE 3:**
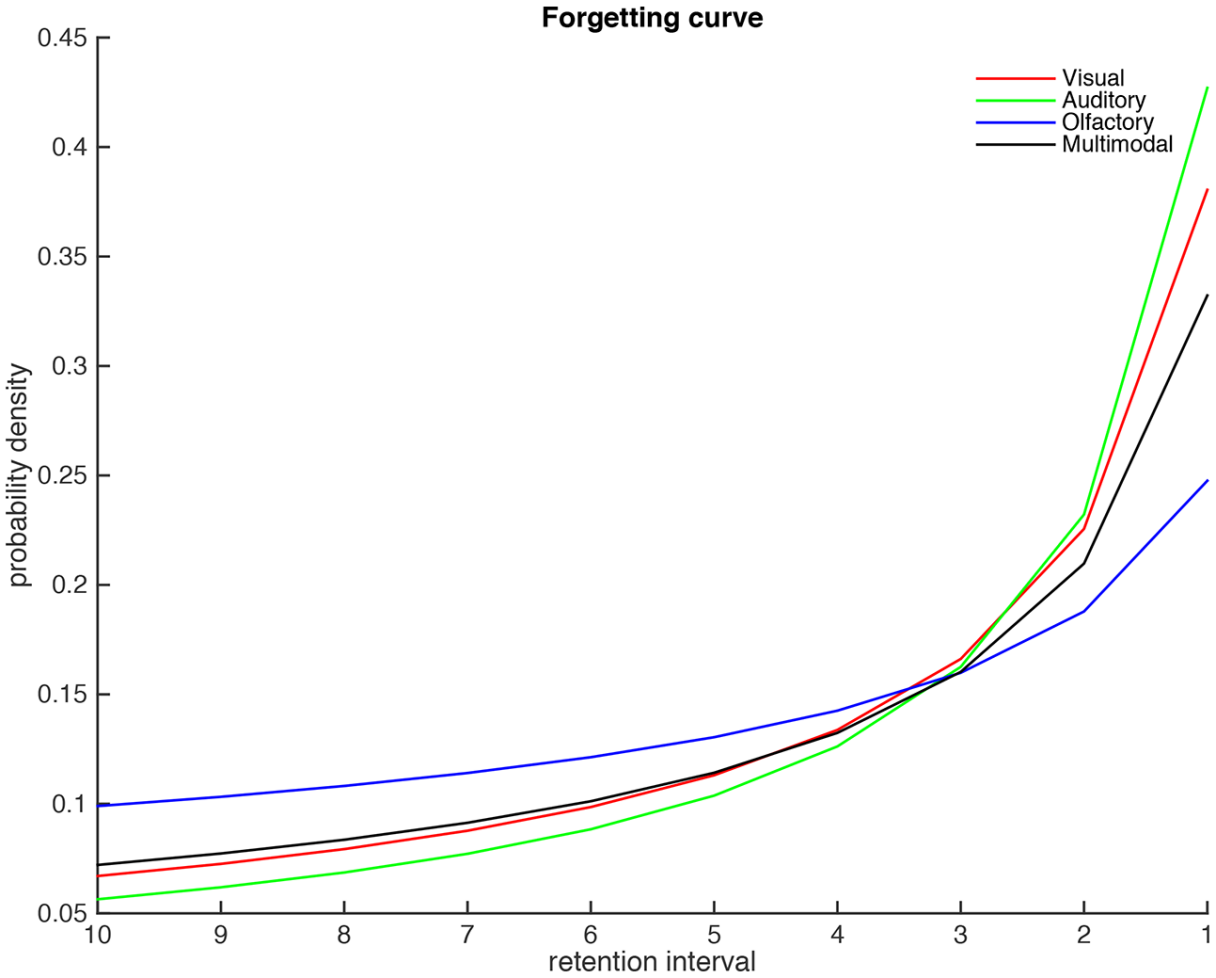
**The forgetting curves of the four conditions**.

The Janssen et al. (2011) procedure also estimates parameters to power-function forgetting curves that are fitted to the 10 most recent years. These curves are plotted in **Figure 3**. The results shows that the visual and auditory modalities are more strongly remembered for the most recent years, but at the same time show a faster forgetting over the years, compared to the olfactory condition [*F*(3,58) = 57.07, *p* < 0.05 η^2^ = 0.125]. This effect is slightly stronger for the auditory compared to the visual modality. In contrast the olfactory condition shows a rather shallow forgetting and a moderate memory for recent memories. Finally, the multimodal condition show intermediate slope and recency effects, although this conditions is more similar to the visual and auditory conditions compared to the olfactory condition. The exponents of the power-function for the visual, auditory, olfactory and the multimodal conditions where –0.74, –0.88, –0.40, and –0.66, respectively, and their associated intercepts where 0.38, 0.43, 0.25, and 0.33, respectively.

### Experiential Ratings

Mean values for the five experiential ratings were calculated for each participant. Next, the mean values were analyzed using five separate one-way ANOVAs with modality as between group factor. The ANOVAs did not yield any significant differences (*p*s > 0.05) across the four modalities. See **Table 3** for further details.

**Table 3:** Mean experiential ratings (and SD) across the four conditions and η^2^ for the five ANOVAs.

	Condition				
	
Experiential rating	Visual	Auditory	Olfactory	Multimodal	η^2^
Pleasantness	3.29 (0.43)	3.52 (0.48)	3.47 (0.54)	3.47 (0.43)	0.037
Feeling of being brought back in time	3.50 (0.57)	3.32 (0.50)	3.58 (0.52)	3.61 (0.47)	0.049
Vividness	3.52 (0.50)	3.48 (0.54)	3.53 (0.60)	3.50 (0.57)	0.001
Importance	2.45 (0.47)	2.40 (0.88)	2.56 (0.68)	2.79 (0.68)	0.049
Emotionality	2.74 (0.31)	2.64 (0.46)	2.83 (0.74)	2.92 (0.64)	0.040


## Discussion

The present study is the first to investigate the age distribution of multimodal cues in autobiographical memory retrieval. Results showed that the age distribution of autobiographical memories varies as a function of cue-modality. In addition to replicating previous findings on the age distributions for visual and olfactory retrieval cues, two novel distributions were also shown, i.e., the age distributions for naturalistic auditory cues (cf. Schulkind et al., 1999; Schulkind and Woldorf, 2005; Cady et al., 2008) and multimodal cues. Importantly, modelling of the age distributions suggests that multimodal is more similar to visual and auditory age distributions than the olfactory distribution, and that the multimodal retrieval of autobiographical memories may be more driven by visual and auditory information. No differences were observed across the retrieval cues regarding experiential ratings or number of retrieved events.

In conjunction with previous research on visual dominance the present study suggest that there is a hierarchy among the modalities contained in a multimodal cue. We suggest based on modelling of the age distributions that visual and auditory information are the dominant sensory input when probing autobiographical memories with multimodal retrieval cues. The findings of the current study are in line with the results of Karlsson et al. (2013). Thus, the suggested modality hierarchy has been demonstrated using two different types of data, i.e., age distribution data and the content of autobiographical memories. Extrapolating from previous studies on visual dominance (e.g., Schmid et al., 2011), it may be speculated that visual information contributes more than auditory information to the retrieval outcome. This view is in line with Greenberg and Rubin (2003) who suggest that the visual system is central for autobiographical memory and that sensory systems can activate each other during autobiographical memory recall in a cascading fashion.

Potentially, the underlying mechanism for this suggested cue-dominance could be related to attentional processing of sensory information. For example, from perception experiments on visual dominance utilizing non-autobiographical protocols it has been suggested that visual dominance is the result of more attention being directed toward visual information compared to information pertaining to other sensory systems (e.g., Posner et al., 1976; Sinnett et al., 2007). Although the present study did not address the underlying mechanism for the suggested cue-dominance we suggest that this effect may be related to an asymmetry in attention occurring either at (a) encoding or (b) at retrieval when sensory input (i.e., the retrieval cue) is processed.

No synergistic effects were observed among the modalities in the multimodal cue. A synergistic effect between visual, olfactory, and auditory information in the multimodal cue would likely have been manifested as a higher frequency of retrieved events, higher experiential ratings, and a unique age distribution (i.e., not being modelled on the three unimodal distributions) for the multimodal condition compared to the unimodal conditions. None of this was the case in the present data. The lack of synergistic effects provides further support for the view that there is a dominating modality (e.g., vision) within the multimodal cues.

Despite the seemingly smaller contribution of olfaction on multimodal retrieval it is possible that olfaction influence multimodal retrieval indirectly by modulating attention. For example, Seo et al. (2010) demonstrated using an eye-tracking protocol that participants were more prone to attend odor-congruent pictures when presented simultaneously with odor-congruent and -incongruent food pictures.

It was also shown that the forgetting curves of the visual, auditory, and multimodal conditions were relatively similar. However, the forgetting curve of olfactory condition was less steep compared to the other three conditions. It could be speculated that forgetting curve of the olfactory condition is in part related to proactive interference. A stronger proactive interference would predict a less steep forgetting curve. The notion of stronger proactive interference in odor memory compared to other modalities and a unique neural representation for first-learned olfactory associations has been supported in several studies (e.g., Lawless and Engen, 1977; Yeshurun et al., 2009). We suggest that the similarity between the forgetting curves of the visual, auditory, and multimodal conditions provide further support for the notion that multimodal retrieval of autobiographical memory is mainly driven by visual and auditory processes.

In line with previous work on childhood amnesia (e.g., Rubin and Schulkind, 1997; Bruce et al., 2005) the present study indicated that few memories were retrieved from the 0–5 years interval. **Figure 3** suggest that odor-evoked memories may have an earlier onset than memories retrieved by pictures or sounds. However, conclusions concerning cue-modality differences with regard to the childhood amnesia would require a follow-up study targeting childhood amnesia specifically.

It should be noted that the participants in the current study represent a young sample (20–30 years). Typically, the age range is around 50 years and upward in age distribution studies. Although the present study did detected differences in the age distributions across cue conditions it is suggested that further studies are carried out with older participants.

Given that several (unimodal) studies have reported differences in experiential ratings (e.g., pleasantness/emotiona lity, vividness, and the feeling of being brought back in time) as a function of cue-modality it is somewhat surprising that no significant differences were observed in the experiential ratings of the present study (cf. Ehrlichman and Bastone, 1992; Chu and Downes, 2002; Herz and Schooler, 2002; Herz, 2004; Goddard et al., 2005; Willander and Larsson, 2006, 2007). However, null hypothesis significance testing does not permit any conclusions regarding null findings and the lack of differences regarding experiential ratings could potentially be related to power. An potential alternative explanation could also be that the effect sizes of the studied variables are weaker in the current study compared to previous work due to, e.g., the age of the participants or sample size. The effect sizes (Cohen’s *d*) typically range between 0 and 1 in previous studies where the phenomenological experiences (valence/emotionality, vividness, feeling of being brought back) of autobiographical memories cued by different modalities were contrasted (e.g., Rubin et al., 1984; Chu and Downes, 2002; Goddard et al., 2005; Willander and Larsson, 2006, 2007). However, in some instances the effect sizes exceed *d* = 3 (Herz and Schooler, 2002; Herz, 2004). Overall these effect sizes from previous work indicated that odor-cued memories are rated as more emotional and associated with stronger feelings of being brought back in time to the occurrence of the event. Regarding vividness the results are less consistent across studies. Based on the effect sizes of the current study the multimodally and olfactory cued memories seems to evoke somewhat stronger phenomenological experiences than the visual and auditory cued events, although these differences were non-significant. Thus, with regard to the magnitude and direction of the effect sizes, it seems as if the present study is in line with previous work. Based on the results of the present study we suggest that experiential ratings could be insufficient and that thorough analyses of different experiential dimensions based on content (i.e., verbal descriptions) and semantic scales are needed as a complement to better understand potential differences across modalities (cf. Karlsson et al., 2013).

In summary, the present study replicated and extended previous findings concerning the age distributions for autobiographical retrieval cues. More importantly, we suggest that there is a modality hierarchy in multimodal retrieval cues, such that multimodal retrieval is mainly driven by visual and auditory information.

## Conflict of Interest Statement

The authors declare that the research was conducted in the absence of any commercial or financial relationships that could be construed as a potential conflict of interest.
