# Supplementary material for: Multimodal retrieval of autobiographical memories: sensory information contributes differently to the recollection of events
Source: Front Psychol. 2015 Nov 5;6:1681. doi: 10.3389/fpsyg.2015.01681 (PMC4633501; doi:10.3389/fpsyg.2015.01681)
Supplement: Supplementary file 1 [file Data_Sheet_1.DOCX]

Appendix A: The visual, auditory and olfactory stimuli grouped by context
